# Successful use of prolonged cefiderocol monotherapy for the treatment of a complex pleural abscess caused by extensively drug-resistant (XDR) *Pseudomonas aeruginosa*

**DOI:** 10.1093/jacamr/dlad103

**Published:** 2023-09-06

**Authors:** Charlotte Brookfield, Emma Fadden, Louise Sweeney

**Affiliations:** Liverpool Clinical Laboratories, Liverpool University Hospitals NHS Foundation Trust, Mount Vernon Street, Liverpool, UK; Critical Care, Liverpool University Hospitals NHS Foundation Trust, Mount Vernon Street, Liverpool, UK; Department of Microbiology, Manchester University NHS Foundation Trust, Oxford Road, Manchester, UK

This case report describes the prolonged use of monotherapy cefiderocol for the treatment of a pulmonary abscess caused by XDR *Pseudomonas aeruginosa*.

A 56-year-old male was admitted to hospital (Day 1) with respiratory failure, 8 days after a positive COVID-19 test. A CT scan demonstrated typical changes associated with COVID-19 pneumonitis. Past medical history included mild COPD, hypertension and previous smoking. Baseline functional status was excellent, and he had received two COVID-19 vaccines. He was admitted to the ICU for continuous positive airway pressure (CPAP) support, alongside our treatment protocol for COVID-19 of sarilumab, dexamethasone and empirical antibiotics (IV amoxicillin/clavulanic acid and oral doxycycline). Deranged liver function tests precluded treatment with remdesivir.

On Day 7, he developed pleuritic left posterior chest wall pain and a repeat CT pulmonary angiogram (CTPA) demonstrated segmental pulmonary embolus (PE), extensive COVID-19-related lung disease and cavitation in the left lower lobe.

On Day 12, *P. aeruginosa* was isolated, innocuously, from a mid-stream urine (MSU). On Day 13, he was found to have a left-sided tension pneumothorax and developed severe cardiovascular and respiratory instability. CT scan demonstrated worsening pneumonitis and progression of cavitation.

On Day 37, a left-sided empyema and pleural abscess developed, from which we isolated *P. aeruginosa*. At this point, the patient had been receiving almost continuous broad-spectrum antibiotic therapy for episodes of sepsis and infective lung changes. Table 1 summarizes the chronological isolation of *P. aeruginosa*, resistance patterns and antibiotic treatment. First-line anti-pseudomonal antibiotic susceptibility testing was performed by disc diffusion, and second-line extended susceptibility testing by broth microdilution (Thermo Scientific™ Sensititre AST system and Gram Negative MDRGNX2F AST plate).

**Table 1.** Summary of clinical isolates of *P. aeruginosa* with sensitivity data and antimicrobial therapy

Sample (day of admission)	CIP	CAZ	TZP	MEM	ATM	CZA	C/T	GEN	AMK	TOB	CST	FDC	Antibiotics at time of sample collection (day of admission antibiotics started)
MSU (12)	I	I	I	S	—	—	—	S	S				
BAL (16)	I	I	I	S	—	—	—	S	S	—	—		MEM, LZD (14, 14)
BAL (24)	I	I	I	S	—	—	—	S	S	—	—		MEM, VAN (14, 18)
Tracheal aspirate (36)	I	R	R	R	R	S	S	S	S	S	S		MEM (14)
Pleural fluid (38)	I	I	I	S	S	S	S	S	S	S	S (2)	S (1)	TZP (38)
	R	R	R	R	R	R	R	R	R	R	S (2)	S (2)	TZP (38)
Tracheal aspirate (38)	I	R	I	R	—	—	—	S	S	—	—		TZP, CIP (38, 40)
Pleural fluid (40)	I	I	I	S	—	—	—	S	S	—	—		TZP, CIP (38, 40)
	I	R	I	R	—	—	—	S	S	—	—		TZP, CIP (38, 40)
Pleural fluid (44)	I	I	I	R	—	—	—	S	S	—	—		TZP, CIP (38, 40)
BAL (68)	R	R	R	R	R	R (16)	S (2)	S	S		S (2)	S (1)	TZP, CIP (38, 40)
Pleural fluid (74)	I	R	I	R				S	S				FDC, nebulized CST (71)
Tracheal aspirate (114)	R	R	R	R			S (1)	S	S			S (2)	FDC stopped Day 114

CIP, ciprofloxacin; CAZ, ceftazidime; TZP, piperacillin/tazobactam; MEM, meropenem; ATM, aztreonam; CZA, ceftazidime/avibactam; C/T, ceftolozane/tazobactam; GEN, gentamicin; AMK, amikacin; TOB, tobramycin; CST, colistin; FDC, cefiderocol; LZD, linezolid; VAN, vancomycin; BAL, bronchoalveolar lavage; S = sensitive at standard dosing, I = sensitive at increased dosing, R = resistant, where available MIC data are recorded in brackets (mg/L).

By Day 68, the patient was colonized with several phenotypically different strains of *P. aeruginosa*, including isolates resistant to ceftazidime/avibactam (MIC > 16 mg/L). MBL gradient test strip (ETEST^®^MBL, bioMérieux) and carbapenemase PCR detection (Cepheid GeneXpert^®^) were performed on many of the isolates but no specific resistance mechanism was detected. Unfortunately, due to a processing error, the isolate was not referred to the reference laboratory for further characterization.

Repeat CT demonstrated bilateral dense consolidation and effusions, with a persisting left-sided pleural abscess. This information, combined with increasing evidence of clinical failure, led to a change of therapy on Day 71 to cefiderocol 2 g IV every 8 h based on creatinine clearance of 79 mL/min^1^ (MICs ranged between 1 and 2 mg/L) in combination with nebulized colistin 2 million units every 8 h. Use of ceftolozane/tazobactam was considered; however, one isolate had tested resistant and, at the time, this antibiotic was unavailable due to supply issues.^1^ The abscess was managed with additional chest drains but a definitive surgical procedure was ruled out due to the likely risk of further complications. On Day 81, colistin was stopped owing to conclusions that it was adding little therapeutic synergy in nebulized form.

A repeat CT on Day 113 of admission showed improvement in the appearances of the empyema, effusions and consolidation. It was agreed that cefiderocol would be discontinued at the point of tracheostomy decannulation, which took place on Day 114, equating to 42 days of treatment with cefiderocol. A tracheal aspirate collected at decannulation demonstrated *P. aeruginosa* with continued susceptibility to cefiderocol. The patient experienced no adverse drug reactions related to his cefiderocol treatment.

The patient was stepped down from critical care to the ward the following day and continued to improve. On Day 127 of admission, he was discharged home with no oxygen requirement.

Cefiderocol is a novel cephalosporin antibiotic with broad Gram-negative bactericidal activity^2^ licensed for the treatment of aerobic Gram-negative infections in patients with limited treatment options.^3^ The unique method of entry into the bacterial cell using iron transport channels in the cell wall protects cefiderocol from some commonly acquired resistance mechanisms.^4^

Given its novelty, there is minimal experience in its use for the treatment of deep and complex infections. Previous case reports concerning longer courses to target XDR *P. aeruginosa* have included successful treatment of an intra-abdominal infection,^5^ chronic osteomyelitis in a child^6^ and aortic valve infective endocarditis.^7^ Others have reported on treatment of a pulmonary empyema in conjunction with dual therapy and surgery^8^ and the development of resistance after 6 weeks treatment for a complex pancreatic abscess.^9^

This case report highlights the safety and durability of monotherapy cefiderocol over a 42 day treatment period in the management of a complex post-COVID-19 pleural empyema from which XDR *P. aeruginosa* was isolated from culture.
